# Effect of Correcting the Presenilin‐1 Point Mutation Using CRISPR‐Cas in Neural Precursors from Patient‐Derived Fibroblasts Through Cellular Reprogramming in Familial Alzheimer's Disease

**DOI:** 10.1002/alz70855_103956

**Published:** 2025-12-23

**Authors:** Jesus‐Adrian Buendia‐Meraz, Maria‐del‐Carmen Silva‐Lucero, Juan‐Ramon Padilla‐Mendoza, Maria‐del‐Carmen Cardenas‐Aguayo

**Affiliations:** ^1^ UNAM, School of Medicine, Department of Physiology, CDMX, DF, Mexico; ^2^ División de Químico Biológicas, Universidad Tecnológica de Tecámac, Mexico, EM, Mexico

## Abstract

**Background:**

Alzheimer's disease (AD) is a neurodegenerative disorder classified as either sporadic or familial (FAD), depending on the genetic component and age of onset. Understanding and applying gene‐editing tools like CRISPR‐Cas is of great relevance for correcting mutations and/or alterations to reverse the pathological phenotype of neurodegenerative diseases.

**Method:**

To achieve this objective, we performed cell culture, immunodetection via Western blot, immunocytofluorescence, and viral transduction.

**Result:**

Cell culture characterization of control and patient‐derived NPCs was performed using immunocytofluorescence and Western blot detection of SOX2, Oct3/4, Nanog, Nestin associated with stem cell stages, as well as cellular maturation states (b‐III‐Tub, GFAP, Olig2), proliferation (Ki67), and differentiation (MAP2, NeuN). We found that FAD‐derived NPCs expressed more mature markers of neuronal differentiation than control individuals' NPCs. Viral transduction with Adeno‐associated viruses (AAV‐9) carrying the necessary gene sequences for mutation correction in patient‐derived cells was successfully performed.

**Conclusion:**

Control patient cells exhibited better characterization towards stem‐like phenotypes, while cells from patients with the FAD mutation of interest showed markers of cellular maturation. Viral transduction was successfully carried out; however, further analysis is needed to determine the restoration of the healthy phenotype.

